# Immediate Root-Filling

**Published:** 1887-07

**Authors:** J. Smith Dodge

**Affiliations:** New York, N. Y.


ARTICLE X.

### IMMEDIATE ROOT-FILLING.

BY J. SMITH DODGE, JR., M. D., D. D. S., NEW YORK, N. Y.

This practice may be familiar to others, but it came to my mind as a direct application of Listerian surgery, and I am not aware that anyone has adopted it outside of my immediate circle. At any rate, the uniform results of about a year's experience lead me to publish my plan.

What is here said relates exclusively to roots from which the dentist removes a portion of living pulp. It may be only that little fibre near the apex which is so slow to die, or it may be any greater amount. But unless the last of the pulp removed gives pain, or (what I like better) followed by a drop of blood, this article has no reference to that root. And if the final bit of root-pulp is taken away alive, it makes no difference what may have been the connection of the rest. When I have a hopeless pulp to get rid of, with immediate root-filling in prospect, I generally make an application of arsenic and wait till the bulk of the pulp is dead. Those who, after removing this, pass a fine broach actually to the apex, will seldom fail to find the desired remnant of life. I have several times disregarded the slight apical soreness which often accompanies the death of the pulp, and have had no bad result.

The process is simply this: Being sure that a shred of living pulp remains, I make everything ready for the entire operation, with full protection of the tooth from saliva. The living shred of pulp is quickly removed; any blood that follows is absorbed by a thread of bibulous paper passed up the canal; the strongest possible solution of carbolic acid is injected into the canal, churned to the apex with a broach, and absorbed with a thread of paper, then a thread of gutta-percha (Hill's stopping) previously prepared and cold is passed as far as posssible into the canal, and with warm wires packed to the very end. When the filling is pressed against the apical constriction it generally gives a little pain, which is satisfactory as showing that the canal is full. When the whole root is filled to the pulp-chamber, it is forever disposed of.

Of course, if there are two or three roots, the same process is applied to all which have canals large enough to permit the manipulations. If any canal is too small for this, it may be freely carbolized and left. I have never, under this or any other treatment, seen trouble from the pulp in a canal which was too small to admit Johanson's broaches.

This finishes the roots. The dentist may do what he likes with the crown.

I have not counted my cases, but they cannot be less than twenty-five or thirty, extending over nine or ten months. Not a single one has had any subsequent trouble. The longest pain was for about an hour, and then absolutely ended. Nearly all have been entirely right by the time the roots were filled.

The plan is based on the well-proved fact that a clean flesh-wound, disinfected and closed from the air, will not suppurate. Abundant experiment has proved this, and all surgeons act on it daily.

To tear away a shred of living pulp leaves at the apex a clean flesh-wound.

If the pulp were alive up to the pulp cavity, I should not care for the carbolic acid after removing. But as the filling material must pass through a part of the canal which may have become infected, it might carry along germs enough to do mischief, and so I fill the canal with the antiseptic.

It is my belief that the habit of waiting a little after the pulp is removed, to see if there will be any trouble, is like those prophecies which are said to fulfill themselves. The waiting brings the trouble.—*Dental Cosmos.*

